# Characterization of the complete mitochondrial genome of the Macaroni penguin *Eudyptes chrysolophus* from the Barton Peninsula, King George Island, Antarctica

**DOI:** 10.1080/23802359.2021.1888329

**Published:** 2021-03-18

**Authors:** Jong-U Kim, Jeong-Hoon Kim

**Affiliations:** Korea Polar Research Institute, Incheon, South Korea

**Keywords:** Crested penguin, *Eudyptes chrysolophus*, Macaroni penguin mitogenome

## Abstract

The Macaroni penguin *Eudyptes chrysolophus* is a small crested penguin. In this study, the complete mitochondrial genome of *E. chrysolophus* is revealed for the first time. The mitogenome sequence is circular and 17,059 bp in length. It contains 13 protein-coding genes (PCGs), 22 transfer RNA (tRNA) genes, and two ribosomal RNA (rRNA) genes similar to other Spheniscidae species. The total nucleotide composition is 30.53% (A), 32.86% (C), 13.96% (G), and 22.66% (T), and 46.81% for overall GC contents. The phylogenetic analysis shows a close relationship between *E. chrysolophus* and *E. schlegeli*. Our findings would be useful for further studies on phylogenetics and evolutionary history of the genus *Eudyptes*.

The Macaroni penguin (*Eudyptes chrysolophus*), a species of crested penguin, is the most abundant penguin species worldwide, distributed from the Antarctic to the Subantarctic regions (Woehler and Poncet 1993). The population of macaroni penguin appears to have declined rapidly over the past three generations (BirdLife International 2020). The taxonomic status of the genus *Eudyptes* and the number of species within it have been repeatedly discussed (Frugone et al. 2018). Complete mitochondrial genomes provide baseline information to understand genomic evolution and facilitate phylogenetic inference (Matsui et al. 2009) and taxonomic clarifications (Sebastian et al. 2018). A mitogenomic study of *E. chrysolophus* have been reported; however, it was incomplete information (Cole et al. 2019). Thus, clarification of complete mitochondrial genome of *E. chrysolophus* is needed to understand their phylogeny and evolution.

Here, we sequenced and analyzed the complete mitogenome of *E. chrysolophus* (GenBank: MW074963). The blood sample (proof number MP1) was obtained at Narebski Point, Antarctica (62°14′14.66″S, 58°46′30.59ʺW), on 10 January 2010, and stored at the Korea Polar Research Institute (KOPRI), Incheon, South Korea. Total genomic DNA was extracted from the blood sample using a DNeasy Blood and Tissue kit (Qiagen, Hilden, Germany). The complete mitochondrial genome was sequenced and annotated according to our previous study (Kim and Kim 2020). An Illumina paired-end (PE) library was prepared according to the manufacturer’s instructions with 550 bp inset size, and sequencing was performed using an Illumina sequencing platform supplied by a commercial company (Phyzen, Seongnam, South Korea).

The complete mitochondrial genome sequence of *E. chrysolophus* had a circular genome of 17,059 bp in length, containing 13 protein-coding genes (PCGs), 22 transfer RNA (tRNA) genes, and two ribosomal RNA (rRNA) genes. The overall base composition is 30.53% (A), 32.86% (C), 13.96% (G), and 22.66% (T). The heavy strand codes 28 genes and the light strand codes 9 genes. The 13 PCGs of *E. chrysolophus* encode 3798 amino acids. Most PCGs started with ATG codon, except for *COI* and *ND3* which use the initiation codon GTG and ATT, respectively.

In order to ascertain the phylogenetic status of *E. chrysolophus*, a phylogenetic analysis was performed comparing the mitogenome of the *E. chrysolophus* with the inclusion of 21 published mitogenomes from the family Spheniscidae species. A phylogenetic tree was constructed by the maximum likelihood (ML) method with 1000 bootstrap replicates, using the MEGA 7.0 program (Kumar et al. 2016) and applying the Tamura–Nei model (Figure 1). The phylogenetic relationship revealed that *E. schlegeli* (MK290250) was the most closely related species to *E. chrysolophus* (MW074963) as a sister group. Although *E. chrysolophus* (MK290242) is already registered at the NCBI as a partial sequence, the results of a phylogenetic analysis based only on PCGs for the same species showed slightly lower similarity compared with *E. schlegeli* (MK290250). We believe it would be worth studying inter-species mutations in further studies.

**Figure 1.**
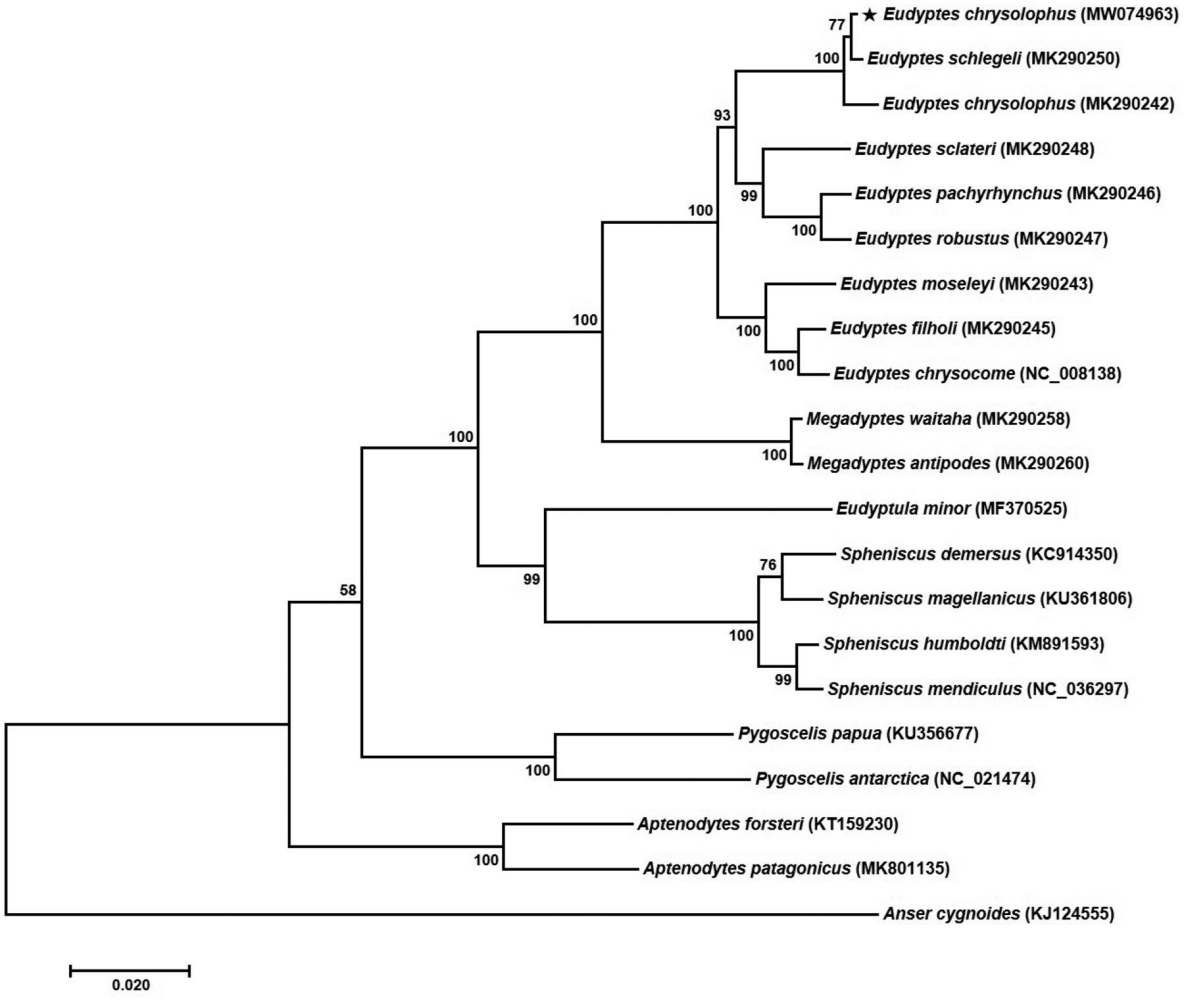
Phylogenetic tree of *Eudyptes chrysolophus* and other Spheniscidae family based on mitochondrial PCGs. The numbers in the nodes indicate bootstrap support values (>50%) from 1000 replicates.

## Data Availability

The genome sequence data that support the findings of this study are openly available in GenBank of NCBI at (https://www.ncbi.nlm.nih.gov/) under the accession no. MW074963. The associated BioProject, SRA, and Bio-Sample numbers are PRJNA667576, SRR12778075, and SAMN16378289 respectively.
